# Targeting the Liquid–Liquid Separation Region of c‐Maf for Treating Chromosomal Translocations in Multiple Myeloma

**DOI:** 10.1002/mco2.70464

**Published:** 2025-11-11

**Authors:** Ze Wang, Mengjie Guo, Xichao Yu, Yi Sun, Haowen Bai, Lianxin Zhou, Zihao Liu, Hongming Huang, Chong Wang, Hong Liu, Chunyan Gu, Ye Yang

**Affiliations:** ^1^ Nanjing Hospital of Chinese Medicine Affiliated With Nanjing University of Chinese Medicine Nanjing China; ^2^ School of Medicine Nanjing University of Chinese Medicine Nanjing China; ^3^ Department of Hematology Affiliated Hospital of Nantong University Nantong China; ^4^ Department of Hematology the First Affiliated Hospital of Zhengzhou University Zhengzhou China

**Keywords:** benzoyl benzoic acid, c‐Maf, chromosomal translocation, liquid–liquid phase separation, multiple myeloma

## Abstract

Multiple myeloma (MM), as an incurable hematologic malignancy, is driven by chromosomal translocations. Recent research suggests that targeting phase separation may offer a new therapeutic vulnerability for MM. The transcription factor c‐Maf is implicated in MM‐associated translocations and undergoes liquid–liquid phase separation (LLPS). Although c‐Maf is historically classified as “undruggable” for its transcription factor nature, we propose a new therapeutic strategy targeting its phase separation mechanism. Comprehensive in vitro and in vivo experiments indicated that elevated c‐Maf promoted MM progression through LLPS. Mechanistically, the alanine‐rich intrinsically disordered regions (IDRs) in c‐Maf drive hydrophobic interactions, enabling phase separation. These IDRs recruit RNA Polymerase II (RNA Pol II) through similar interactions, forming oncogenic condensates that activate the Mtbp/c‐Myc axis. Notably, we discovered benzoyl benzoic acid (BBA) as a candidate compound targeting the inter‐IDR interaction domain of c‐Maf. BBA binding impedes c‐Maf's LLPS capacity, consequently blocking RNA Pol II recruitment and Mtbp transcriptional activation. This mechanistic interference successfully inhibited c‐Myc‐driven MM progression in experimental models. Our study demonstrates that c‐Maf‐driven LLPS activates the c‐Maf/RNA Pol II/Mtbp/c‐Myc axis, highlighting phase separation targeting as a promising therapeutic strategy for translocation‐associated MM patients.

## Graphical abstract

Targeting the liquid‐liquid separation region of c‐Maf for treating pathogenic chromosomal translocations in multiple myeloma. c‐Maf promotes cell proliferation by forming liquid–liquid phase separation (LLPS) to recruit RNA Pol II and activate the Mtbp/c‐Myc axis in multiple myeloma (MM), while targeting the LLPS region of c‐Maf impedes the progression of MM.

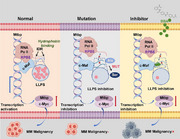

## 1 Introduction

While contemporary treatment modalities have significantly improved clinical outcomes for multiple myeloma (MM) patients, achieving durable remission remains elusive as most patients inevitably experience disease relapse and eventual therapeutic failure [1]. One of the main obstacles in eliminating MM is its significant genetic complexity, specifically the high occurrence of chromosomal translocations in approximately 50% of cases that drive malignant transformation by disrupting normal cell cycle progression [2]. While recognized as critical oncogenic drivers, these pathogenic chromosomal rearrangements present formidable technical challenges for direct genomic correction. Of particular clinical significance is the ectopic positioning of proto‐oncogenes within active chromosomal loci, which induces their pathological overexpression, a molecular mechanism that fuels tumorigenesis, disease advancement, and acquired drug resistance [3]. This pathobiological framework highlights the critical imperative to pioneer mechanism‐driven approaches that both decode the oncogenic drivers encoded by these genetic aberrations and engineer molecularly targeted agents to counteract their tumorigenic signaling cascades, ultimately creating novel therapeutic avenues for MM patients with treatment‐refractory chromosomal lesions [3].

Liquid–liquid phase separation (LLPS) is a crucial physicochemical process that has gained significant attention in recent years due to its role in organizing various cellular processes by forming biomolecular condensates [4]. Growing evidence implicates dysregulated LLPS dynamics in cancer pathogenesis, particularly through aberrant interactions with oncogenic pathways [5]. In particular, LLPS is linked to oncogenes involved in chromosomal translocations. Notably, pharmacological targeting of the NSD2‐mediated SRC‐3 LLPS process enhances sensitivity to bortezomib (BTZ) therapy in MM [6]. Furthermore, Myc‐driven LLPS exerts selective transcriptomic modulation that promotes malignant proliferation [7]. In MM pathogenesis, primary chromosomal translocations predominantly involve partners such as CCND1, FGFR3/MMSET, MAF, CCND3, and MAFB, while secondary translocations frequently affect MYC, MAP3K14, NFKB1, IL16, IL2RA, IKBKE, TXNDC5, NSD2, and members of the cyclin D family (CCND1‐3) [8, 9]. Our systematic analysis using the PhaSePred database to evaluate the LLPS potential of these translocation‐associated proteins revealed c‐Maf as the highest‐ranking candidate, suggesting its central role in phase separation‐mediated oncogenesis.

Previous studies on c‐Maf as a potential treatment for MM have primarily focused on inhibiting posttranslational modifications and altering upstream/downstream signaling pathways. However, these approaches exhibit limited specificity, as they may inadvertently affect other Maf family proteins, including MafA and MafB, due to structural and functional conservation within this transcription factor (TFs) family [10, 11]. While transcription factors (TFs) are classified as “undruggable” targets due to their lack of conventional binding pockets [12], emerging strategies highlight the therapeutic potential of targeting non‐DNA‐binding domain (non‐DBD) regions. The regions of TFs outside of the DNA‐binding domains (DBDs), known as non‐DBD regions, are often enriched with intrinsically disordered regions (IDRs). They guide TFs to their genomic binding sites and have the potential to undergo LLPS [13]. Recent advances in targeting LLPS capabilities of oncogenic TFs have spurred interest in developing selective small‐molecule inhibitors [14]. Therefore, further research on the IDRs of c‐Maf and their involvement in LLPS could aid in the development of targeted small‐molecule inhibitors for c‐Maf.

In this study, we examined the correlation between c‐Maf's ability to undergo LLPS and the progression of MM. Furthermore, we investigated the mechanisms underlying c‐Maf's impact on transcriptomic changes through LLPS. Notably, we identified benzoyl benzoic acid (BBA) as a novel LLPS inhibitor of c‐Maf and evaluated its effectiveness through both in vitro and in vivo experiments.

## 2 Results

### 2.1 c‐Maf Undergoes LLPS in Both In Vitro and In Vivo Experiments

**FIGURE 1:**
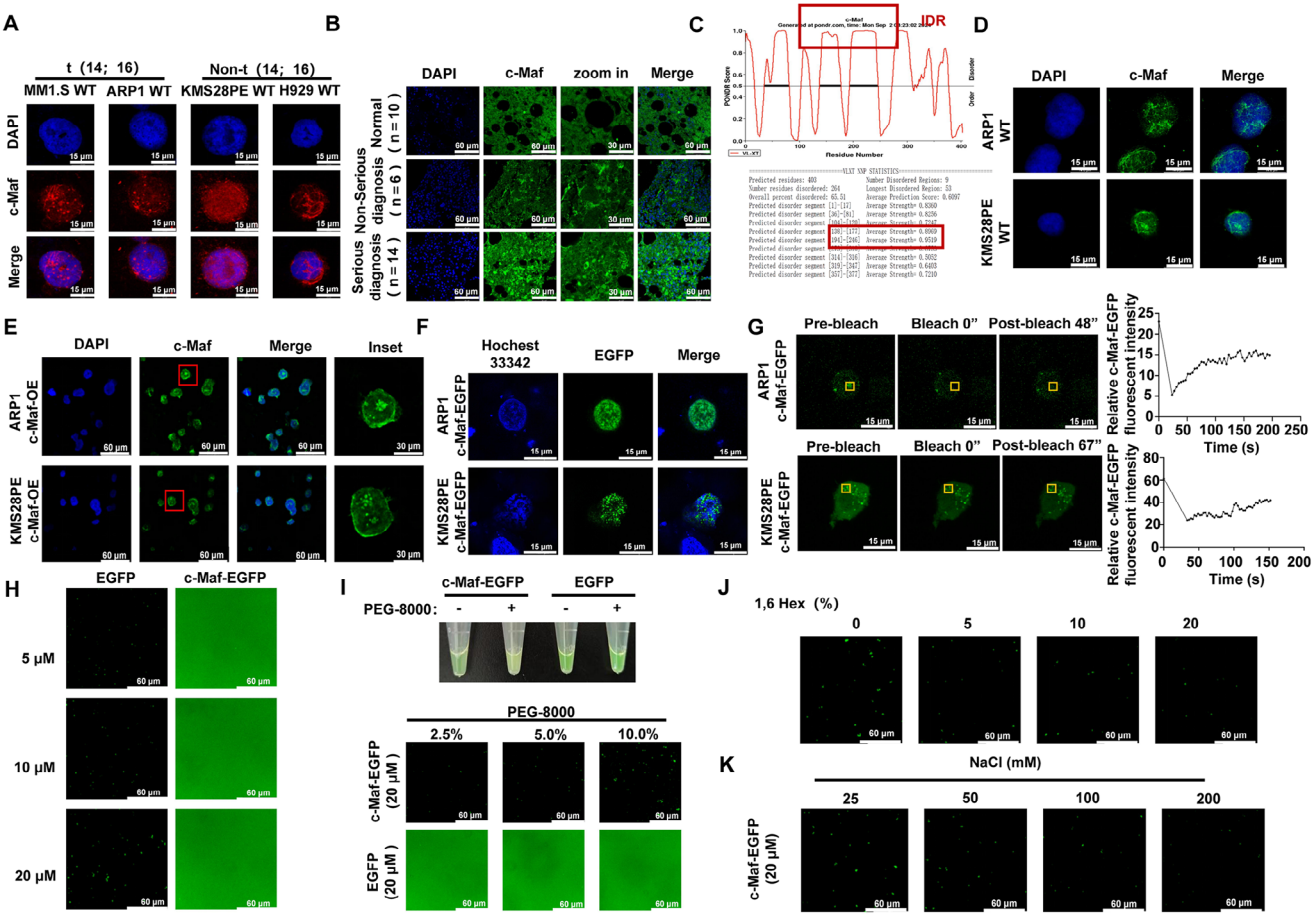
c‐Maf undergoes LLPS in both in vitro and in vivo experiments. Immunofluorescence (IF) assay was used to measure c‐Maf expression in *t*(14;16) MM cell lines (MM1.S and ARP1) and non *t*(14;16) MM cell lines (KMS28PE and H929). Scale bar: 15 µm. (B) Representative IF staining was performed on normal (*n* = 10), nonserious diagnosis (*n* = 6), and serious diagnosis (*n* = 14) MM patient samples. Scale bar: 60 µm. (C) The PONDR database for phase‐separating proteins was used to predict the IDRs of c‐Maf protein. A higher score indicates a greater probability of the position being an IDR, with 0.5 serving as the boundary for whether it is an IDR. (D) Confocal microscope images were captured to show the spot‐like distribution of c‐Maf in the nucleus of WT MM cells. Scale bar: 15 µm. (E) Representative confocal images of c‐Maf cellular distribution showed that c‐Maf‐OE cells had a more remarkable discrete speckle‐like distribution. Scale bar: 60 µm. (F) Live cell imaging observed the typical spot‐like distribution of c‐Maf in MM cell lines transfecting with c‐Maf‐EGFP. Scale bar: 15 µm. (G) The Fluorescence Recovery After Photobleaching (FRAP) assay was used to measure the kinetic recovery times of c‐Maf‐EGFP bleached droplet foci in ARP1 and KMS28PE MM cell lines. Scale bar: 15 µm. (H) Representative images of droplet formation of c‐Maf‐EGFP and EGFP with different protein concentrations. Scale bar: 60 µm. (I) Representative images of droplet formation of c‐Maf‐EGFP and EGFP with a protein concentration of 20 µM before and after the addition of PEG‐8000 to final concentrations of 2.5%, 5%, and 10%. Scale bar: 60 µm. (J) Representative images of droplet formation of c‐Maf‐EGFP with a protein concentration of 20 µM before and after the addition of 1,6‐hexanediol to final concentrations of 5%, 10% and 20%. Scale bar: 60 µm. (K) Representative images of droplet formation of c‐Maf‐EGFP with a protein concentration of 20 µM before and after treatment with different concentrations of NaCl. Scale bar: 60 µm. The data are expressed as the mean ± SD; *p < 0.05, **p < 0.01, and ***p < 0.001.

The PhaSePred tool was utilized to assess the possibility of LLPS formation among the key proteins involved in chromosomal translocation of MM. As shown in Table 1, c‐Maf was identified as having the highest LLPS‐self score. MM patient cohorts, combined with MTT assay and apoptosis analysis, provided the evidence that c‐Maf acts as a driver gene for MM malignancy (Figure S1). IF (Immunofluorescent) staining revealed a higher concentration of c‐Maf protein in the nucleus of *t*(14;16) MM cell lines compared to non‐*t*(14;16) cell lines (Figure 1A). Further analysis of MM patient samples also showed a more pronounced distribution of c‐Maf speckles in patients with a serious initial diagnosis compared to those with a nonserious initial diagnosis or normal individuals (Figure 1B and Figure S1F). Additionally, analysis of c‐Maf using PONDR, NovoPro, and IUred2a databases revealed the presence of large IDRs in the 138–177 and 194–246 amino acid regions, which are known to contribute to LLPS formation (Figure 1C and Figure S1J,K).

**TABLE 1:** The possibility of LLPS formation among the main proteins involved in chromosomal translocation of MM by analyzing PhaSePred database.

Uniport entry	Entry name	Gene name	LLPS‐self Score (proteins that can adopt LLPS spontaneously in vitro)
O75444	MAF_HUMAN	MAF	0.787 (top 4.991%)
P01106	MYC_HUMAN	MYC BHLHE39	0.767 (top 5.919%)
P19838	NFKB1_HUMAN	NFKB1	0.716 (top 7.966%)
Q14005	IL16_HUMAN	IL16	0.606 (top 11.661%)
O96028	NSD2_HUMAN	NSD2 KIAA1090 MMSET TRX5 WHSC1	0.604 (top 11.73%)
Q9Y5Q3	MAFB_HUMAN	MAFB KRML	0.318 (top 24.211%)
P30281	CCND3_HUMAN	CCND3	0.187 (top 36.731%)
P24385	CCND1_HUMAN	CCND1 BCL1 PRAD1	0.160 (top 41.163%)
Q14164	IKKE_HUMAN	IKBKE IKKE IKKI KIAA0151	0.134 (top 46.798%)
P22607	FGFR3_HUMAN	FGFR3 JTK4	0.134 (top 46.655%)
Q16548	B2LA1_HUMAN	BCL2A1 BCL2L5 BFL1 GRS HBPA1	0.111 (top 56.903%)
Q8NBS9	TXND5_HUMAN	TXNDC5 TLP46 UNQ364/PRO700	0.107 (top 59.44%)
Q99558	M3K14_HUMAN	MAP3K14 NIK	0.100 (top 63.455%)
P29965	CD40LG CD40L TNFSF5 TRAP		0.099 (top 63.951%)
P01589	IL2RA_HUMAN	IL2RA	0.075 (top 80.977%)
P30279	CCND2_HUMAN	CCND2	0.065 (top 90.587%)

Abbreviations: LLPS, liquid–liquid phase separation; MM, multiple myeloma.

To further confirm the aggregation of c‐Maf protein and its association with LLPS properties, we utilized standard LLPS validation techniques both in vitro and in vivo. IF staining showed that c‐Maf‐overexpression (c‐Maf‐OE) MM cells displayed a more distinct speckle‐like distribution compared to wild‐type (WT) cells (Figure 1D,E). Live cell imaging also revealed the typical spot‐like distribution of c‐Maf in ARP1 and KMS28PE cells transfected with c‐Maf‐EGFP (Figure 1F). FRAP assay demonstrated that c‐Maf exhibited rapid fluorescence recovery within seconds, indicating its ability to drive droplet dynamics (Figure 1G). We also performed in vitro droplet assays and observed that purified recombinant c‐Maf‐EGFP protein formed droplets in a concentration‐dependent manner, unlike the control protein EGFP (Figure 1H). When we added the purified recombinant c‐Maf‐EGFP protein to buffers containing 10% PEG‐8000, the c‐Maf‐EGFP solution became opaque due to droplet aggregation, while the EGFP solution remained clear (Figure 1I). Additionally, the size and distribution of c‐Maf‐EGFP protein droplets were significantly reduced with increasing concentrations of 1,6‐hexanediol (Figure 1J) and NaCl (Figure 1K), respectively. These results provide strong evidence that c‐Maf is capable of forming LLPS in MM.

### 2.2 The Absence or Modification of IDRs Impedes the Cancer‐Causing Potential of c‐Maf by Obstructing Its Ability to Undergo LLPS in MM

**FIGURE 2:**
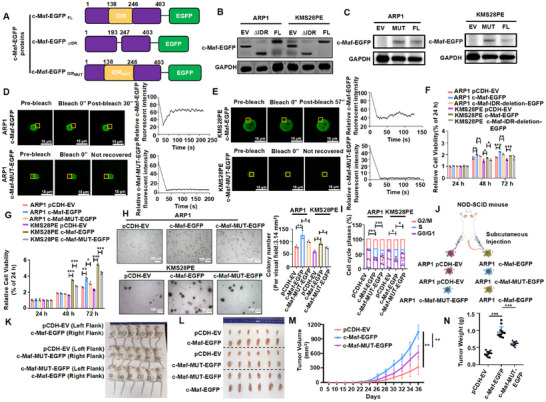
The absence or modification of IDRs impedes the cancer‐causing potential of c‐Maf by obstructing its ability to undergo LLPS in MM. (A) Three plasmids were designed for EGFP fusion protein, deletion, and mutation (alanine to serine mutation) based on the two segments of c‐Maf IDRs with the highest scores. (B) WB method confirmed the successful construction of stable transgenic cell lines with c‐Maf IDR deletion. (C) WB method confirmed the successful construction of stable transgenic MM cell lines with c‐Maf IDR mutations (alanine to serine). (D, E) The kinetic recovery times of bleached c‐Maf‐IDR‐mutation‐EGFP (c‐Maf‐MUT‐EGFP) droplet foci were compared in ARP1 (D) and KMS28PE (E) cell lines using the FRAP assay, in comparison to c‐Maf‐EGFP. Scale bar: 15 µm. (F) CCK‐8 assay revealed a significant increase in cell proliferation in ARP1 and KMS28PE cell lines following transfection with c‐Maf‐EGFP compared to EV‐transfected cells, while the absence of IDRs impaired this pro‐proliferative effect. (G) CCK‐8 experiment showed that transfection with c‐Maf‐MUT‐EGFP weakened pro‐proliferative effect of c‐Maf on MM cell lines. (H) Soft agar cloning experiment proved that induction of c‐Maf promoted long‐term proliferation of MM cell lines, while mutation of c‐Maf‐IDRs suppressed it. The field of view area value is 3.14 mm^2^. (I) Cell cycle assay showed that the proportion of S phase of c‐Maf‐EGFP‐transfected cells was significantly increased compared to EV‐transfected cells, which was decreased by transfection with c‐Maf‐MUT‐EGFP. (J) Subcutaneous xenograft tumor mouse model. (K) Photographic images of mice injected with MM cells transfected with pCDH‐EV, c‐Maf‐EGFP, and c‐Maf‐MUT‐EGFP in each flank. (L) Photographic images of tumors. (M, N) The time course of tumor volume (M) and mean tumor weight (N) from three experimental groups at Day 36 after implantation of the specified MM cells. The data are expressed as the mean ± SD; *p < 0.05, **p < 0.01, and ***p < 0.001. EV, empty vector; FL, full length; MM, multiple myeloma; WB, western blotting.

We investigated the factors that influence the ability of c‐Maf to undergo LLPS. It was found that IDRs of c‐Maf are enriched in hydrophobic amino acids, particularly alanine (Figure S1L). To further explore the role of alanine in the IDRs for the LLPS ability of c‐Maf, we introduced mutations that replaced alanine with the hydrophilic and uncharged amino acid serine. To achieve this, we designed three plasmids for EGFP fusion protein, deletion, and mutation (Figure 2A), which were successfully transfected into MM cell lines and validated by WB assay (Figure 2B,C). To confirm the significance of alanine in the IDRs for the LLPS ability of c‐Maf, we performed a FRAP assay and observed that the c‐Maf‐IDR‐mutation‐EGFP (c‐Maf‐MUT‐EGFP) protein was unable to recover its fluorescence intensity before quenching (Figure 2D,E).

Furthermore, we explored the impact of disrupting c‐Maf IDRs on MM malignancy. MM cells transfected with c‐Maf‐IDR‐deletion‐EGFP or c‐Maf‐MUT‐EGFP exhibited significantly lower cell growth rates compared to those transfected with c‐Maf‐EGFP (Figure 2F,G). In addition, colony formation assay showed that c‐Maf‐EGFP‐transfected MM cells exhibited a significant increase in long‐term cell growth, which was significantly reduced by c‐Maf‐MUT‐EGFP (Figure 2H). Cell cycle analysis also demonstrated that c‐Maf‐MUT cells had a decreased proportion in the S phase compared to c‐Maf‐EGFP cells (Figure 2I). To extend these findings in vivo, we subcutaneously injected ARP1 cells with pCDH‐EV, c‐Maf‐EGFP, and c‐Maf‐MUT‐EGFP into the NOD‐SCID mice (Figure 2J). After 28 days, we observed that tumors in the c‐Maf‐EGFP group grew faster than those in the EV group (Figure 2K,L). The mean volume and weight of c‐Maf‐EGFP tumors were significantly higher than EV tumors (Figure 2M,N). In contrast, the tumorigenicity induced by c‐Maf was obviously hindered upon c‐Maf mutation (Figure 2M,N). Our findings suggest that c‐Maf IDRs, particularly the alanine residues within them, are responsible for its ability to undergo LLPS and its oncogenic function in MM.

### 2.3 c‐Maf Increases the Expression of Mtbp Contributing to the Progression of MM Malignancy

**FIGURE 3:**
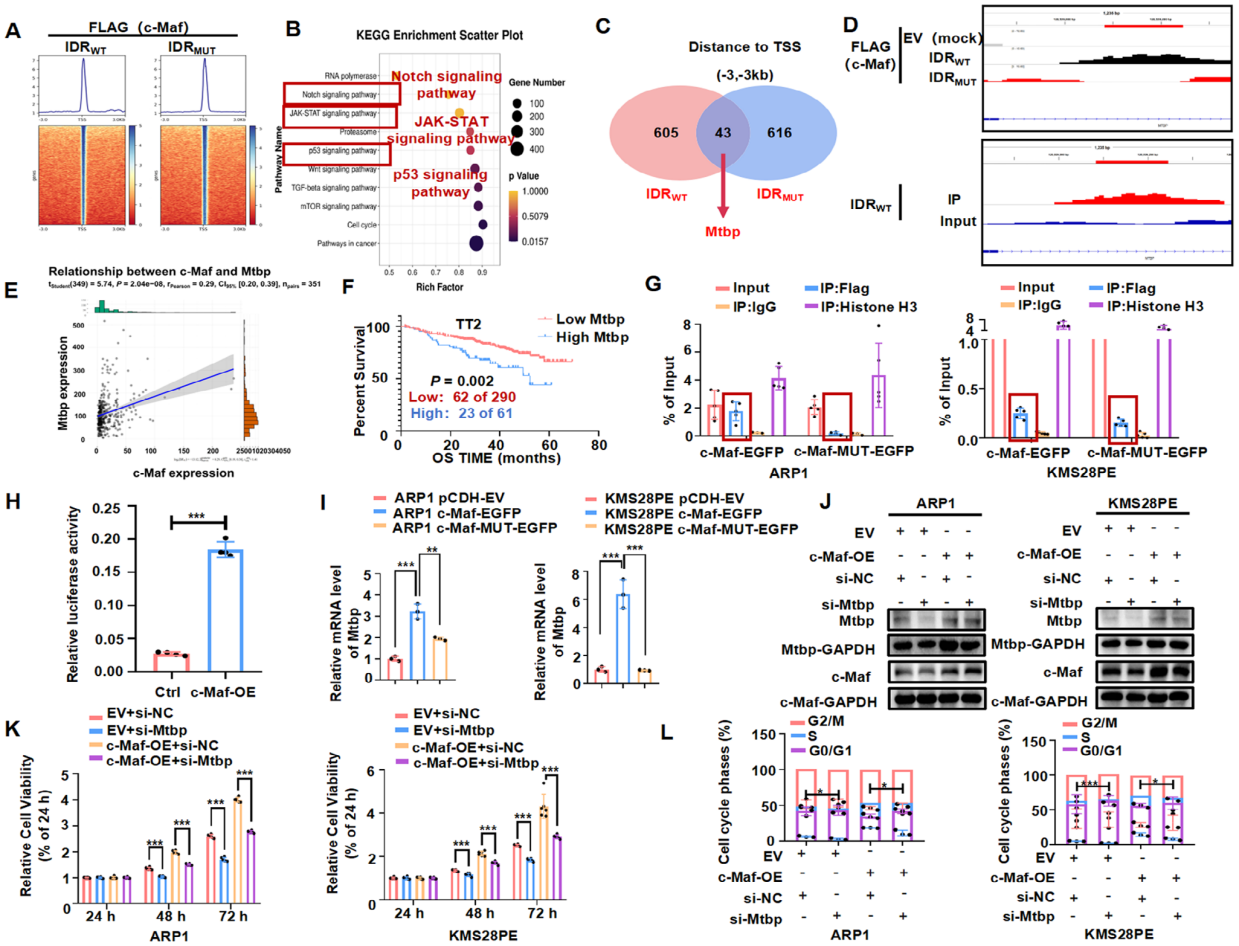
c‐Maf increases the expression of Mtbp contributing to the progression of MM malignancy. (A) ChIP‐seq was performed on ARP1 cells that were transfected with c‐Maf‐EGFP and c‐Maf‐MUT‐EGFP, using the Flag antibody as bait. (B) KEGG pathway enrichment analysis of ChIP‐seq. (C) Mtbp was identified through a Venn diagram analysis of the upregulated and downregulated genes in two groups, as well as KEGG pathway enrichment analysis. (D) Peak enrichment map of c‐Maf on Mtbp gene shows that IDR mutations significantly decreases the binding abundance of c‐Maf and Mtbp promoters. (E) The analysis of TT2 patient cohort showed a positive correlation between the expression of Mtbp and c‐Maf in newly diagnosed patients (*n* = 351). (F) The analysis of TT2 database revealed a significant correlation between elevated levels of Mtbp and decreased overall survival in MM patients. (G) ChIP‐qPCR assay confirmed Flag c‐Maf binds to the promoter region of Mtbp in ARP1 and KMS28PE cell lines. (H) Co‐transfection of c‐Maf‐OE plasmid significantly increased the fluorescence intensity of firefly luciferase in HEK293 cells, as shown by the dual luciferase reporter gene experiment. (I) RT‐qPCR experiments showed that Mtbp expression was upregulated upon c‐Maf overexpression and downregulated upon c‐Maf mutation. (J) WB examination confirmed that silencing Mtbp resulted in a downregulation of its protein expression level, while overexpressed c‐Maf upregulated its expression level. (K) CCK‐8 assay showed that inhibiting Mtbp hindered the growth of both WT and c‐Maf‐OE MM cells. (L) Cell cycle assay revealed that interfering with Mtbp significantly reduced the proportion of cells in the S phase in both WT and c‐Maf‐OE MM cells. The data are expressed as the mean ± SD; *p < 0.05, **p < 0.01, and ***p < 0.001. EV, empty vector; MM, multiple myeloma; OS, overall survival; WB, western blotting; WT, wild‐type.

To explore the potential targets of c‐Maf, we performed chromatin immunoprecipitation and next‐generation sequencing (ChIP‐seq) in ARP1 cells transfected with c‐Maf‐EGFP and c‐Maf‐MUT‐EGFP plasmids. Through analyses of KEGG and Venn diagrams, we identified Mtbp, DDX6, and ELP4 as potential candidate targets (Figure 3A–C). We then used ChIP‐seq and MM GEP cohorts to screen c‐Maf downstream targets, which were upregulated following c‐Maf overexpression and positively correlated with MM progression. Then, Mtbp was identified as the potential target, since the mRNA expression levels of DDX6 and ELP4 remained unchanged following c‐Maf overexpression (Figure S2A). In addition, peak enrichment map analysis showed that the binding abundance of c‐Maf to the Mtbp promoter was decreased by c‐Maf mutation (Figure 3D). The analysis of the TT2 database indicated a significant positive correlation between Mtbp and c‐Maf expressions in newly diagnosed patients (*n* = 351) (*R* ^2 ^= 0.29, *p *< 0.001) (Figure 3E). Furthermore, increased expression of Mtbp was associated with worse overall survival (OS) in the TT2 cohort (Figure 3F). These findings suggest that Mtbp may serve as a potential target of c‐Maf in MM.

The ChIP‐qPCR experiment showed that c‐Maf binds to the promoter region of Mtbp to activate transcription, which was also further confirmed by an agarose gel electrophoresis experiment (Figure 3G and Figure S2B). In contrast, the mutated form of c‐Maf (c‐Maf‐MUT) repressed this binding. These results were also supported by a dual luciferase reporter gene analysis (Figure 3H). Additionally, an increase in Mtbp mRNA levels was observed upon c‐Maf induction compared to cells transfected with empty vector (EV), which was suppressed by c‐Maf mutation (Figure 3I). Accordingly, enforced c‐Maf expression significantly increased the protein levels of Mtbp (Figure 3J and Figure S2C). These findings confirm that c‐Maf IDRs induce Mtbp transcription, leading to an upregulation of Mtbp protein levels. To investigate the role of Mtbp in MM, siRNA was used to knock down Mtbp in both WT and c‐Maf‐OE MM cells, confirmed by WB examination (Figure 3J and Figure S2C). CCK‐8 assay and cell cycle distribution analyses showed that silencing Mtbp significantly inhibited MM cell proliferation in both WT and c‐Maf‐OE MM cells compared to control cells (Figure 3K,L). These results suggest that c‐Maf forms LLPS to promote MM progression by upregulating Mtbp expression.

### 2.4 c‐Maf Recruits RNA Polymerase II, Leading to LLPS‐Like Behaviors and Promoting Mtbp Transcription

**FIGURE 4:**
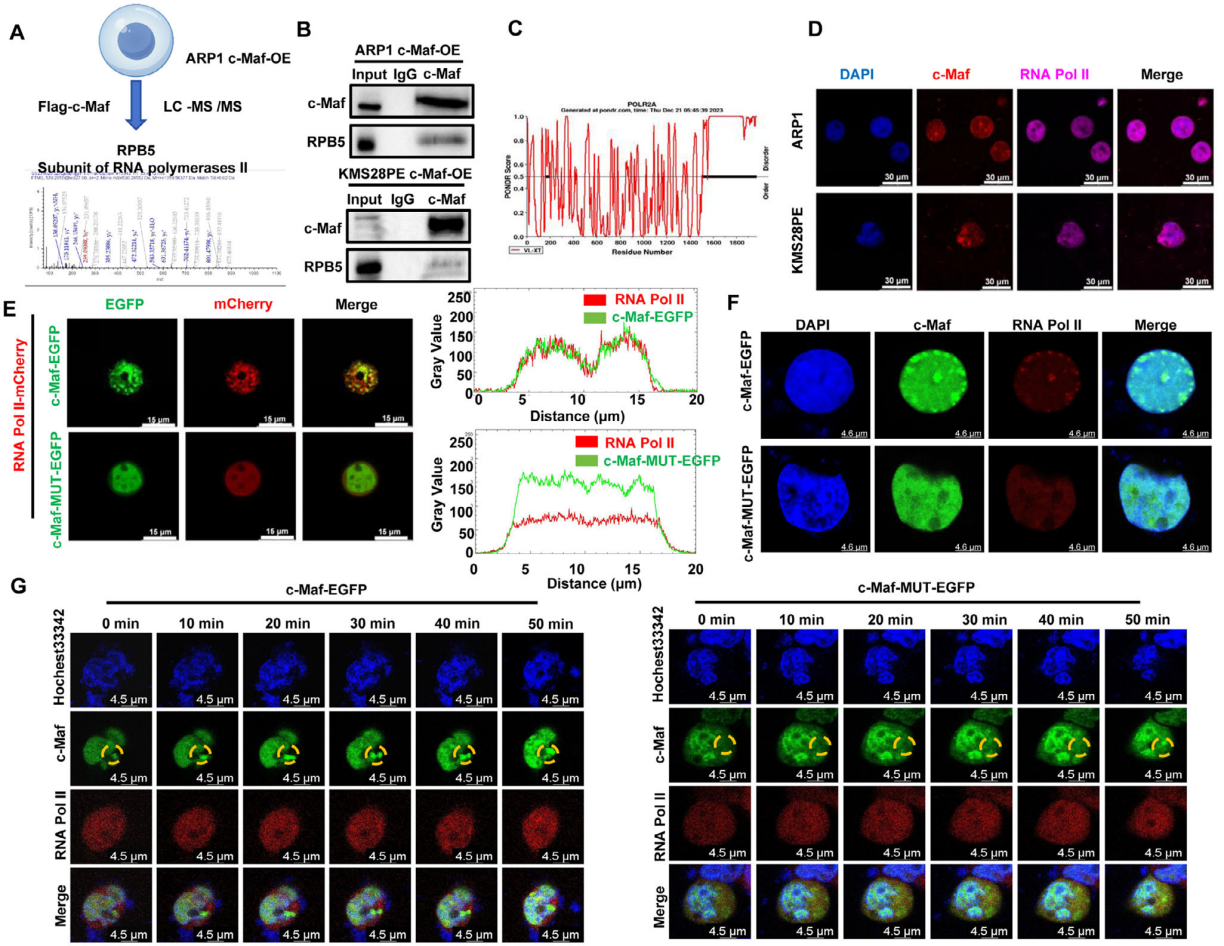
c‐Maf recruits RNA Pol II, leading to LLPS‐like behaviors and promoting MTBP transcription. Mass spectrum (MS) analysis performed on ARP1 c‐Maf‐OE cells identified the specific peptide fragments of multiple subunits of RNA Pol II, especially RPB5 subunit. (B) Co‐IP experiment showed that c‐Maf can interact with RPB5 in both MM cell lines. (C) Analysis of PONDR database revealed the presence of IDR at the C‐terminus of RNA Pol II (POLR2A). (D) IF assay showed that c‐Maf and RNA Pol II co‐localized in the nucleus of both MM cell lines. Scale bar: 30 µm. (E) Live cell imaging experiments showed that there was no scattered pattern of LLPS in the distribution of c‐Maf and RNA Pol II upon mutation of c‐Maf IDRs. Scale bar: 15 µm. (F) Comparison of c‐Maf‐EGFP/RNA Pol II‐mCherry co‐localization before and after mutation of c‐Maf IDRs. Scale bar: 4.5 µm. (G) Dynamic changes in co‐localization of c‐Maf‐EGFP/RNA Pol II‐mCherry before and after mutation of c‐Maf IDRs. Scale bar: 4.5 µm. MM, multiple myeloma; MS, Mass spectrometry.

High concentrations of RNA Polymerase II (RNA Pol II), TFs, coactivators, and mediators are able to form dynamic phase‐separated condensates via multivalent interaction to facilitate gene transcription [15]. Thus, we further explored the potential transcription‐related factors involved in the regulation of c‐Maf on Mtbp transcription through LLPS. We performed mass spectrometry (MS), experiment on ARP1 c‐Maf‐OE cells and identified the specific peptide fragments of multiple subunits of RNA Pol II, including RPB5, RPB8, and RPB7.6, with RPB5 being the largest subunit (Figure 4A). Co‐IP assay confirmed a strong interaction between c‐Maf and RPB5 (Figure 4B). Additionally, analysis of the PONDR database revealed the presence of an IDR structure at the C‐terminus of RNA Pol II (POLR2A) (Figure 4C), suggesting that RNA Pol II may possess LLPS properties. IF analysis showed co‐localization of c‐Maf and RNA Pol II in the nucleus of ARP1 and KMS28PE cells (Figure 4D). Live cell imaging experiments in HEK293 cells further demonstrated that c‐Maf could recruit RNA Pol II to form droplets, which were disrupted by c‐Maf mutation (Figure 4E). Analysis of static and dynamic variations in c‐Maf‐EGFP/RNA Pol II co‐localization in MM cells also showed similar trends (Figure 4F,G). These findings suggest that c‐Maf IDRs mediate its interaction with RNA Pol II to form transcriptional condensates, thereby activating Mtbp transcription.

### 2.5 c‐Maf Plays a Role in the Progression of MM Through the Mtbp‐Mediated c‐Myc Signaling Pathway

**FIGURE 5:**
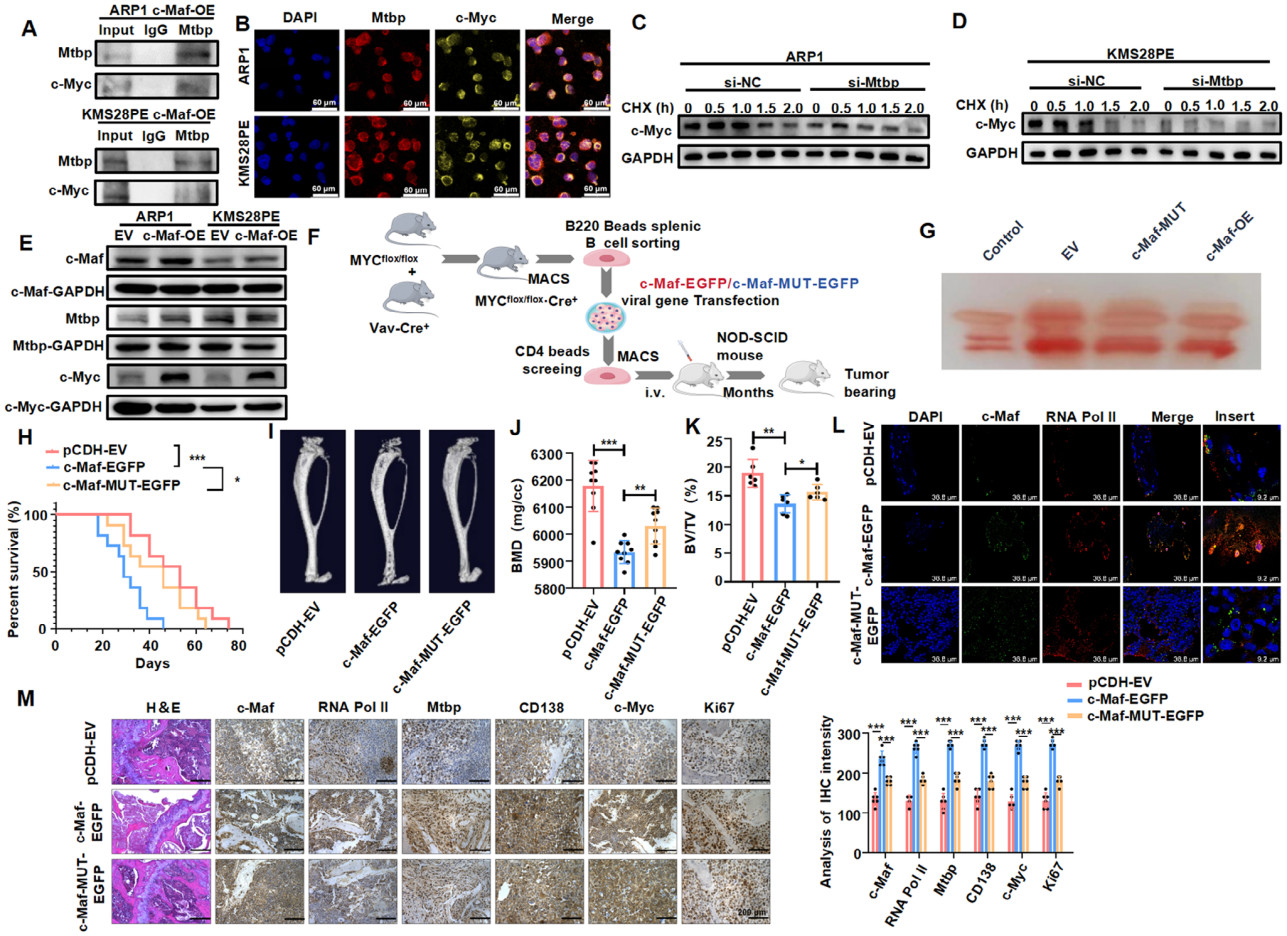
c‐Maf plays a role in the progression of MM through the Mtbp‐mediated c‐Myc signaling pathway. Co‐IP experiment demonstrated that Mtbp can interact with c‐Myc in both MM cell lines. (B) IF experiments revealed that Mtbp and c‐Myc co‐localize in the nucleus of both MM cell lines. (C, D) Inhibiting Mtbp reduced the stability of c‐Myc protein in ARP1 (C) and KMS28PE (D) cell lines. (E) WB examination showed that c‐Maf overexpression upregulated the protein expression levels of Mtbp and c‐Myc. (F) A schematic representation of doptive B cell implantation mouse model. (G) The serum protein electropherogram showed significant M‐spikes (monoclonal Ig) in mice with plasma cell neoplasms (PCNs). (H) The survival times of mice injected with c‐Maf‐MUT‐EGFP transfected B cells were significantly extended compared to the c‐Maf‐EGFP group. (I) MicroCT 3D images of bones in the EV, c‐Maf‐EGFP, and c‐Maf‐MUT‐EGFP groups. (J) BMD of doptive B cell implantation mice in the EV, c‐Maf‐EGFP, and c‐Maf‐MUT‐EGFP groups. (K) BV/TV of doptive B cell implantation mice in the EV, c‐Maf‐EGFP, and c‐Maf‐MUT‐EGFP groups. (L) IF assay showed a significant increase in co‐localization of c‐Maf and RNA Pol II in the c‐Maf‐EGFP group compared to EV group, while mutaion of c‐Maf impaired this co‐localization. Scale bar: 36.8 µm. (M) HE staining of histological sections of the bones in EV, c‐Maf‐EGFP, and c‐Maf‐MUT‐EGFP groups. IHC analysis showed the expression of c‐Maf, RNA Pol II, Mtbp, CD138, c‐Myc, and Ki67 in EV, c‐Maf‐EGFP, and c‐Maf‐MUT‐EGFP groups. Scale bar: 200 µm. The data are expressed as the mean ± SD; *p < 0.05, **p < 0.01, and ***p < 0.001. BMD, bone mineral density; BV/TV, bone volume; IHC, immunohistochemistry; WB, western blotting.

To investigate the potential impact of the c‐Maf/RNA Pol II/Mtbp axis on typical oncogenic pathways involved in MM progression, we conducted a thorough analysis of the KEGG pathway database using ChIP‐seq data. We found that the differentially expressed genes upon c‐Maf mutation were primarily involved in c‐Myc related signaling pathways in leukemia, including Notch, JAK‐STAT, and P53 pathways [16, 17, 18] (Figure 3B). This is consistent with previous research that has identified c‐Myc dysregulation as a key feature in the genetic landscape of MM [19]. We then conducted a Co‐IP assay to confirm the interaction between Mtbp and c‐Myc (Figure 5A). IF staining analysis showed that Mtbp and c‐Myc are co‐localized in the nucleus (Figure 5B). WB analyses indicated that interfering with Mtbp significantly decreased the stability of c‐Myc protein (Figure 5C,D and Figure S2D). Importantly, WB detection revealed that that overexpression of c‐Maf significantly increased the expression of both Mtbp and c‐Myc (Figure 5E and Figure S2E). These findings provide initial evidence that c‐Myc is a potential downstream target of the c‐Maf/RNA Pol II/Mtbp axis.

We created a mouse model for adoptive B cell implantation using Myc‐Cre mice and investigated the role of the c‐Maf/RNA Pol II/Mtbp/c‐Myc axis in the development of MM in vivo (Figure 5F). The serum of mice with plasma cell neoplasms (PCNs) exhibited a high level of paraproteins, known as M‐spikes, which indicate monoclonal expansions of immunoglobulin‐producing plasma cells (Figure 5G). Kaplan–Meier survival curve analysis showed that the mice in c‐Maf‐EGFP group had a significantly shorter survival period compared to those in EV group (Figure 5H). MicroCT (µCT) imaging also revealed more severe bone damage in c‐Maf‐EGFP group, with decreased bone mineral density (BMD) and bone volume (BV/TV) compared to EV group (Figure 5I–K), which was further confirmed by HE staining (Figure 5M). Intriguingly, the shortened survival time and bone damage caused by c‐Maf‐EGFP were significantly improved by c‐Maf mutation (Figure 5H–K). Immunohistochemistry (IHC) staining showed increased levels of c‐Maf, CD138, and Ki67 proteins in c‐Maf‐EGFP group compared to EV group (Figure 5M), which were downregulated by c‐Maf mutation. These findings suggest that c‐Maf can exacerbate the progression of MM in Myc‐Cre mice through its IDRs, indicating a positive correlation between c‐Maf and c‐Myc. Further investigation in vivo confirmed the involvement of the c‐Maf/RNA Pol II/Mtbp/c‐Myc axis. IF analyses confirmed that overexpression of c‐Maf promoted the co‐localization of c‐Maf and RNA Pol II, which was alleviated by c‐Maf mutation (Figure 5L). IHC staining also revealed increased levels of RNA Pol II, Mtbp, and c‐Myc upon overexpression of c‐Maf, which were decreased by c‐Maf mutation (Figure 5M). Similar results were observed in the CDX model (Figure S2F,G). These findings support the role of c‐Maf in regulating Mtbp/c‐Myc and promoting MM progression through the recruitment of RNA Pol II to form LLPS in vivo.

### 2.6 BBA Is Identified as Specifically Targeting c‐Maf to Impede Its Ability to Undergo LLPS

**FIGURE 6:**
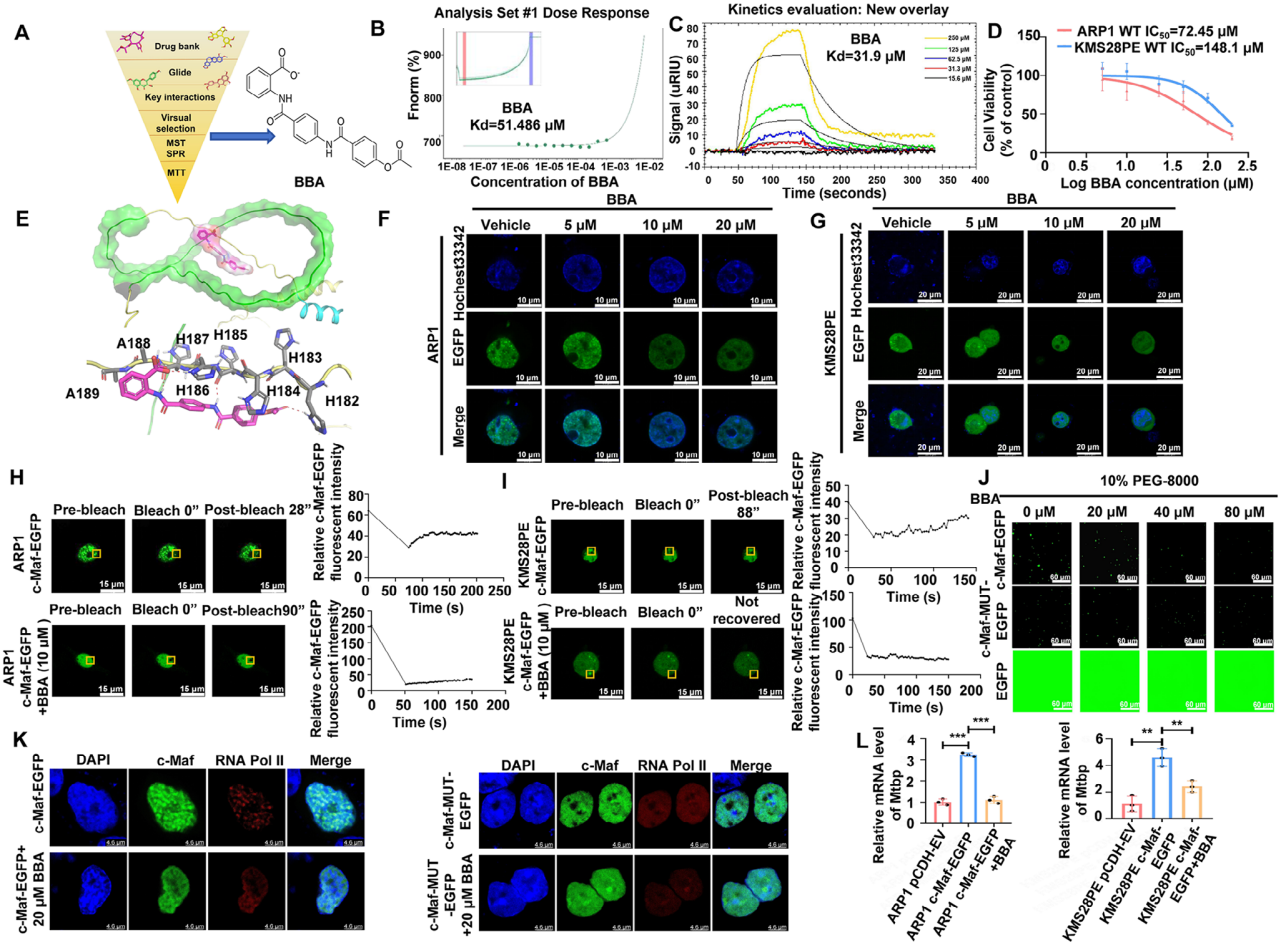
BBA is identified as specifically targeting c‐Maf to impede its ability to undergo LLPS. (A) Workflow of structure‐based virtual screening (left) and chemical structure of hit compound BBA (right). (B) Microscale thermophoresis (MST) experiments demonstrated that BBA has a strong binding affinity with c‐Maf protein. (C) Surface plasmon resonance (SPR) experiments revealed that BBA has the strongest binding affinity with c‐Maf protein among the six selected compounds. (D) MTT experiments showed that BBA has a significant inhibitory effect on MM cells. (E) An overview of BBA binding to c‐Maf is shown, with a detailed binding mode of compound BBA (shown in magenta sticks) binding with c‐Maf. Key residues are shown in grey sticks, and hydrogen bonds are shown in red dotted lines. (F, G) Live cell imaging assays showed that the inhibitory effect of BBA on the LLPS ability of c‐Maf protein is concentration‐dependent in ARP1 (F) and KMS28PE (G) cell lines. Scale bar: 10 and 20 µm. (H, I) FRAP assays found that BBA can inhibit the ability of c‐Maf protein to form LLPS in ARP1 (H) and KMS28PE (I) cell lines. Scale bar: 15 µm. (J) Analysis of the effect of BBA intervention on the formation of LLPS by c‐Maf protein in vitro. Scale bar: 60 µm. (K) Observation of droplet size formed by c‐Maf protein and its co‐localization with RNA Pol II protein in vivo using laser confocal microscopy, when treated with BBA. (L) RT‐qPCR experiments showed that treatment with BBA significantly reduced Mtbp mRNA levels in c‐Maf‐OE MM cell lines. The data are expressed as the mean ± SD; *p < 0.05, **p < 0.01 and ***p < 0.001.

To explore the potential of LLPS as a new approach for pharmacological modulation of the undruggable c‐Maf protein, we conducted a screening for LLPS inhibitors of c‐Maf. We identified two IDRs of the c‐Maf protein (amino acid sequences 138–177 and 194–246) as potential candidates for LLPS (Figure 1C) and predicted potential binding sites within the c‐Maf protein (Figure S3A). Using a structure‐based virtual screening method, we identified six compounds with high affinity for c‐Maf (Figure 6A). Through comparison of microscale thermophoresis (MST), surface plasmon resonance (SPR) and MTT analyses of the six compounds, BBA was identified as a promising inhibitor of c‐Maf (Table 2). Notably, BBA demonstrated a strong binding affinity, with a dissociation constant (Kd) of 51.486 and 31.9 µM in MST and SPR experiments, respectively (Figure 6B,C). Further evaluation showed that BBA significantly suppressed the proliferation of MM cells (ARP1, IC_50_ = 72.45 µM; KMS28PE, IC_50_ = 148.1 µM) (Figure 6D). We then analyzed the binding site of c‐Maf in detail and found that the carboxyl group of BBA engaged in hydrogen and ionic bonds with histidine 186 (H186), while the amide group formed hydrogen bonds with the backbone nitrogen of histidines 187 (H187) and 185 (H185), and the ester group interacted with histidine 182 (H182) (Figure 6E). Interestingly, unlike other LLPS inhibitors that directly targeting the IDRs, the binding site of c‐Maf with BBA was strategically positioned between the two key IDRs located in the LLPS formation region.

**TABLE 2:** Validation of six compounds screened out based on c‐Maf protein structure.

ID	MTT	MST	SPR
Isophthalimide	871.7 µM	416.82 µM	2.27 mM
Difluorophenylurea	533.7 µM	265.95 µM	3.04 mM
Trifluoromethylbenzamide	Unstable	508.96 µM	127 µM
Chlorophenylacetamide	Unstable	555.41 µM	3.31 mM
Dichlorophenylacetamide	260.6 µM	0.12464 µM	1.14 mM
Benzoyl benzoic acid (BBA)	72.45 µM	51.486 µM	31.9 µM

Abbreviations: BBA, benzoyl benzoic acid; MST, microScale Thermophoresis; SPR, surface plasmon resonance.

Moreover, we verified the effect of BBA on disrupting the LLPS ability of c‐Maf. Live cell imaging showed that as the concentration of BBA increased, the punctas of c‐Maf in the nuclei decreased significantly (Figure 6F,G). This was further supported by photobleaching experiments, where BBA‐treated condensates did not show a recovery of c‐Maf fluorescence in KMS28PE cells, and the recovery time was significantly extended in ARP1 cells (Figure 6H,I). In vitro droplet assays also demonstrated that BBA inhibited the formation of spherical droplets of c‐Maf in a concentration‐dependent manner (Figure 6J). Importantly, we observed that BBA did not suppress the expression of c‐Maf (Figure S3B,C). These results indicate that BBA can inhibit the LLPS formation of c‐Maf without affecting its expression. Furthermore, we investigated whether BBA could affect the c‐Maf/RNA Pol II/Mtbp/c‐Myc axis, which is driven by c‐Maf LLPS. IF assays showed that BBA significantly repressed the LLPS formation of c‐Maf and its co‐localization with RNA Pol II, while the inhibitory effect of BBA was significantly reduced after mutation of IDR of c‐Maf (Figure 6K). Consequently, treatment with BBA reversed the upregulation of Mtbp mRNA levels by c‐Maf (Figure 6L). These data demonstrate that BBA binds to the LLPS regions of c‐Maf, disrupting the transcriptional condensates formed by c‐Maf and RNA Pol II, thereby suppressing downstream gene Mtbp transcription.

### 2.7 The Combination of BBA and BTZ Hampers the Growth of MM Cells and Significantly Suppresses MM Tumor Growth

**FIGURE 7:**
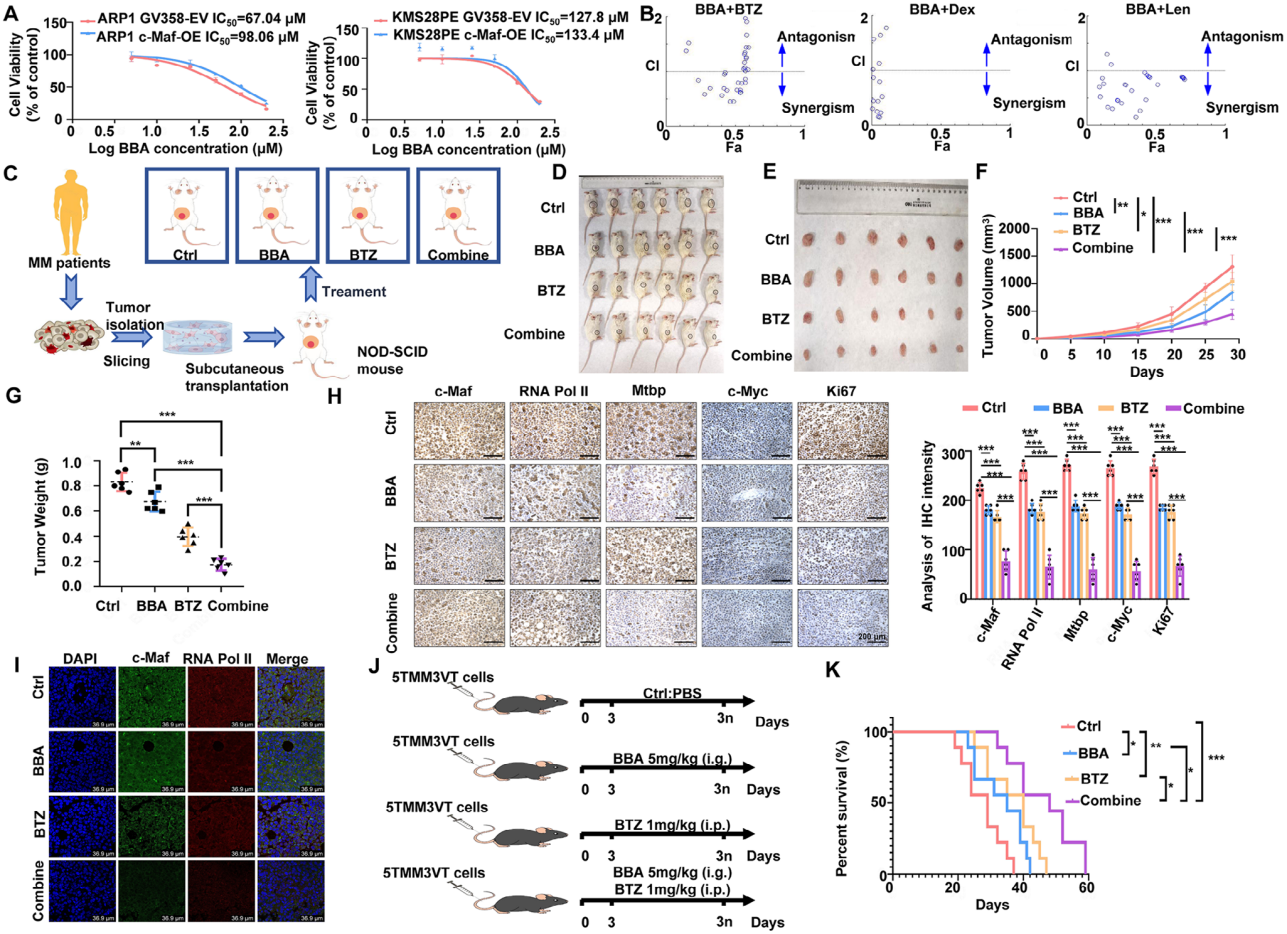
The combination of BBA and BTZ hampers the growth of MM cells and significantly suppresses MM tumor growth. MTT detection examined the effect of different concentrations of BBA on the activity of WT and c‐Maf‐OE MM cell lines. (B) The efficacy of BBA in combination with clinical MM treatment drugs BTZ, Dex, and Len was evaluated using MTT method. (C) Schematic representation of PDX model. (D, E) Photographic images of PDX model mice on Day 30 (D) and tumors taken from mice in each group (E) are shown. (F, G) Tumor growth curves (F) and tumor weight (G) of PDX model mice in the Ctrl, BBA, BTZ, and combination groups. (H) IHC analysis showed that the expressions of c‐Maf, RNA Pol II, Mtbp, c‐Myc, and Ki67 were significantly inhibited in the BBA, BTZ, and combination groups compared to Ctrl group. (I) IF assay showed impaired co‐localization of c‐Maf and RNA Pol II in the BBA, BTZ, and combination groups compared to Ctrl group. (J) Schematic representation of 5TMM3VT mouse model. (K) Treatment with BBA, BTZ, and their combination significantly improved the survival time of 5TMM3VT mice. The data are expressed as the mean ± SD; *p < 0.05, **p < 0.01, and ***p < 0.001. BTZ, bortezomib; Dex, dexamethasone; IHC, immunohistochemistry; Len, lenalidomide; PDX, patient‐derived tumor xenograft; WT, wild‐type.

Finally, we examined the therapeutic potential of BBA in MM. MTT assay showed that BBA inhibited MM cell proliferation, with no significant effect on c‐Maf‐OE MM cells. The IC_50_ of BBA in ARP1 and KMS28PE cells confirmed a stronger effect on the *t*(14;16) MM cell line (ARP1) compared to non‐*t*(14;16) cell line (KMS28PE) (Figure 7A). Then, BBA was combined with typical MM clinical treatment drugs such as BTZ, dexamethasone (Dex), and lenalidomide (Len) to explore whether the combination could enhance their pharmacological activities. MTT assay showed a synergistic effect of BBA, BTZ, and Len, with a combination index (CI) value of less than 1 (Figure 7B). The synergistic effect of BBA and BTZ was further evaluated in vivo using a myeloma PDX model (Figure 7C). Treatment with BBA at a dose of 5 mg/kg significantly inhibited xenograft tumor growth compared to the non‐treatment group (Figure 7D,E). Furthermore, the combination treatment of BBA and BTZ (1 mg/kg) resulted in smaller tumors compared to the single treatment group (Figure 7D,G). IHC and IF experiments confirmed that the elevated expressions of c‐Maf, Ki67, RNA Pol II, Mtbp, and c‐Myc, as well as the co‐localization of c‐Maf and RNA Pol II proteins, were inhibited by treating with BBA, BTZ and their combination, respectively (Figure 7H,I). Notably, the combination treatment achieved the best inhibitory effect (Figure 7H,I). The 5TMM3VT mouse model was also employed (Figure 7J). The combination of BBA and BTZ significantly extended the survival of 5TMM3VT mice, with a superior effect compared to single treatment (Figure 7K). Collectively, the data above demonstrate that targeting c‐Maf with BBA can slow the development of MM in vitro and in vivo, and enhance the anti‐MM effect of BTZ.

## 3 Discussion

The translocation of *t*(14;16)(q32;q23) drives MM pathogenesis by dysregulating c‐Maf through rearrangement, detected in ∼6% of newly diagnosed patients with ∼25% malignant plasma cell involvement. c‐Maf overexpression occurs in ∼50% of myeloma cases, establishing it as a critical oncogenic driver [20]. Of approximately 300 TFs implicated in MM pathogenesis, few have yielded successful small‐molecule therapies [21]. This challenge primarily stems from fundamental structural characteristics of many TFs, including c‐Maf, which typically exhibit intrinsic disorder and lack well‐defined binding pockets, key features required for conventional drug development strategies [22]. Emerging insights from phase separation biology reveal new therapeutic opportunities. Transcriptional condensates formed through LLPS demonstrate selective enrichment of TF‐targeting compounds, suggesting mechanisms to enhance therapeutic specificity while reducing off‐target effects [23]. Modulation of LLPS mechanisms governing TF functionality may enable novel approaches to disrupt pathogenic transcription networks [24]. However, clinical translation of small molecules exploiting this strategy remains nascent.

We conducted a systematic investigation into the role of LLPS in the pathogenesis of MM, while also screening for small molecules that could disrupt c‐Maf‐mediated phase separation. Our comprehensive pharmacological screening led us to identify specific small‐molecule compounds that can modulate c‐Maf activity by selectively disrupting its LLPS functionality. While previous studies emphasized aromatic residue‐mediated interactions in LLPS formation [25, 26], our work revealed the critical contribution of aliphatic hydrophobic residues. Bioinformatic analysis demonstrated that c‐Maf's IDRs exhibit distinct chemical properties, with a significant enrichment of aliphatic residues, particularly alanine clusters (Figure S1L). Alanine emerges as the predominant hydrophobic side chain, exhibiting low‐energy clustering to form hydrophobic patches [27]. The hydrophobic effect drives compact single‐chain conformations in LLPS condensates, enhancing structural stability and dispersion resistance to promote phase separation [28]. As the most stabilizing residue in helical cores, alanine also facilitates intermolecular contacts and modulates droplet material properties, synergizing with multivalent interactions to enhance LLPS capacity by regulating protein fluidity [29, 30]. While aromatic residues like phenylalanine and tyrosine have been extensively studied in LLPS [26, 31, 32, 33], the role of aliphatic hydrophobics remains uncharacterized.

Our investigation revealed that alanine residues critically contribute to the LLPS of c‐Maf, as evidenced by the significant disruption of phase separation upon substituting these residues with hydrophilic serine in the IDRs. This finding establishes alanine enrichment as a potential molecular determinant for LLPS propensity in proteins, while simultaneously proposing that targeted modulation of alanine residues could serve as a strategic approach for probing LLPS mechanisms in intrinsically disordered proteins (IDPs). TFs orchestrate gene activation through the formation of transcriptional condensates, which recruit RNA Pol II, mediator complexes, and cofactors via LLPS [34]. Our MS analysis identified RNA Pol II as a key component recruited by c‐Maf to nucleate these condensates. Among the various nuclear phase‐separated compartments, transcription condensates regulate pre‐initiation complex assembly, transcription initiation, and RNA Pol II phosphorylation [35, 36, 37]. Although the weak multivalent interactions between TFs and RNA Pol II are not well understood, our findings demonstrate that alanine mutations in c‐Maf disrupt its ability to recruit RNA Pol II through disrupted hydrophobic interactions, the primary driving force for this recruitment, consistent with the tyrosine‐mediated hydrophobic contacts governing RNA Pol II LLPS [38]. As a result, this impairment reduces the binding of c‐Maf to downstream targets such as Mtbp, leading to inhibition of transcriptional activation.

We identified two critical activation domains in c‐Maf (aa 138–177 and 194–246; Figure 1C). Unlike conventional strategies that target IDR [39], the small molecule BBA uniquely disrupts inter‐domain interactions at the LLPS interface (aa 182–189). This innovative mechanism selectively suppresses c‐Maf LLPS dynamics by impeding hydrophobic interactions with RNA Pol II, thereby inhibiting Mtbp transcription and c‐Myc‐driven MM progression. The mechanistic congruence between BBA and alanine mutation effects validates the 138–246 aa region as a strategic therapeutic target. BBA's direct targeting of c‐Maf transcriptional activity contrasts with existing inhibitors that indirectly modulate c‐Maf through regulatory partners [10, 11, 20, 40]. Notably, BBA overcomes c‐Maf‐mediated proteasomal inhibitor resistance, showing synergistic efficacy with BTZ in improving survival in a mouse model of MM [41]. While these findings establish LLPS modulation as a promising therapeutic paradigm for “undruggable” TFs, BBA exhibits limitations typical of early‐stage compounds, including suboptimal selectivity [42, 43]. Ongoing medicinal chemistry optimization aims to enhance affinity and specificity, advancing BBA towards lead compound development for MM therapeutics.

Collectively, our integrated analysis delineates how c‐Maf orchestrates the pathogenesis of MM through LLPS. Mechanistically, c‐Maf recruits RNA Pol II via LLPS to establish transcriptional condensates that upregulate Mtbp transcription. This transcriptional reprogramming perpetuates c‐Myc‐driven oncogenesis in MM. Critically, we characterize BBA as a compound disrupting the c‐Maf/RNA Pol II/Mtbp/c‐Myc axis. Our findings demonstrate that BBA has preclinical efficacy in suppressing MM proliferation and tumorigenesis by impairing c‐Maf's phase separation. These findings establish a framework for targeting pathological transcriptional condensates in *t*(14;16) MM, a high‐risk molecular subtype with treatment‐refractory behavior.

## 4 Materials and Methods

### 4.1 Database Analysis

The gene expression profiles of MM patients were obtained from the analysis of two cohorts of patients: total Treatment 2 (TT2, GSE2658) and total Treatment 3 (TT3, GSE2658). These cohorts are available from the GEO database, as previously mentioned [44]. The expression of c‐Maf in different types of clinical diseases and MM cell lines was analyzed using The Human Protein Atlas database (https://www.proteinatlas.org/). The data from the GEO database and The Human Protein Atlas database have obtained the necessary ethical approval. Additionally, the LLPS ability of the c‐Maf protein was assessed using PhaSePred, Natural Disordered Regions (PONDR), NovoPro phase separation, and Iupred2a databases, based on the c‐Maf protein sequence provided by NCBI. PhaSePred is a centralized resource that integrates scores from multiple LLPS‐related prediction tools [45]. The PONDR, NovoPro, and Iupred2a databases are traditional phase separation prediction platforms that use amino acid sequences to predict the LLPS capability of proteins.

### 4.2 MS Analysis

The protein was separated using SDS‐PAGE and the desired gel band was excised and digested using sequencing‐grade trypsin from Promega (USA). The resulting peptide segments were then analyzed using a QExactive mass spectrometer from Thermo Fisher Scientific. The fragment spectrum was compared to the National Center for Biotechnology Information non‐redundant protein database. The original contributions presented in the study are publicly available and can be accessed through the Proteome Xchange Consortium at PXD051828. The MS data can be found at the following URL: https://www.iprox.cn/page/PSV023.html;?url=1725163077932tu0Q.

### 4.3 IF Staining and Confocal Microscopy

Immunofluorescence staining was performed according to the method previously described [46]. Images were captured using a confocal microscope (TCS SP8, Leica, Germany). Samples of MM patients used for IF staining were collected from Jiangsu Provincial Hospital of Traditional Chinese Medicine. This study was approved by the Ethics Committee of Jiangsu Provincial Hospital of Traditional Chinese Medicine (Approval number: 2021NL‐010‐03).

### 4.4 Chromatin Immunoprecipitation and Next‐Generation Sequencing

Cell culture cross‐linking and sample preparation were conducted following the manufacturer's protocol (Chromatin IP Kit, CST, #9003). The ChIP‐seq analysis was performed by Lc‐Bio Technologies (Hangzhou, China) Co, Ltd., and the raw data were deposited in GEO (GSE264392). The ChIP‐seq data can be accessed at the following URL: https://www.ncbi.nlm.nih.gov/geo/query/acc.cgi?&acc=GSE264392. ChIP‐qPCR was carried out using the Chromatin IP Kit (Magnetic Beads) and validated by RT‐qPCR. The amount of immunoprecipitated DNA in each sample was used as a signal to represent the total amount of input chromatin.

### 4.5 Adoptive B‐Cell Transfer Mouse Model

The spleen cells of Myc‐Cre mice were sorted using B220 magnetic beads and then transfected with c‐Maf‐EGFP and c‐Maf‐MUT‐EGFP lentivirus. The transferred B cells successfully engraft and result in PCNs in immunocompetent hosts [47, 48]. The cells transfected with pCDH EV virus were used as blank controls. The successfully transfected B cells (2 × 10^6^ cells/mouse) were injected into NOD‐SCID receptor mice through the tail vein. The incidence of disease was observed every 2 days, and the survival period of the mice was recorded one week after modeling. After the modeling was completed, bilateral tibia samples were collected from each group of mice for Micro CT imaging and IHC staining to detect bone destruction and plasma cell proliferation.

All animal experiments were conducted according to a protocol approved by the Institutional Ethics Review Boards of Nanjing University of Chinese Medicine, in accordance with the Guidelines (Ethics Registration no. 202205A007).

### 4.6 Statistical Analysis

All data were presented as the mean ± standard deviation. To determine the significance between experimental groups, a two‐tailed student's *t*‐test (for two groups) and one‐way ANOVA (for multiple comparisons) were employed. The survival rate of MM patients was determined using the Kaplan–Meier method and Log‐rank test. Statistically significant differences were indicated by *p < 0.05, **p < 0.01 and ***p < 0.001.

## Author Contributions

C.G. and Y.Y. conceived the study. Z.W., X.Y., Y.S., L.Z., H.B. and Z.L. performed the experimental work. Z.W., M.G., X.Y. and Y.S. analyzed the data. M.G. drafted the manuscript. C.W., H.H., H.L., C.G. and Y.Y. revised the manuscript. All authors have read and approved the final manuscript.

## Conflicts of Interest

The authors declare no conflicts of interest.

## Ethics Statement

Samples of MM patients used for IF staining were collected from Jiangsu Provincial Hospital of Traditional Chinese Medicine. This study was approved by the Ethics Committee of Jiangsu Provincial Hospital of Traditional Chinese Medicine (Approval number: 2021NL‐010‐03). The data from the GEO database and The Human Protein Atlas database have obtained the necessary ethical approval. All animal experiments were conducted according to a protocol approved by the Institutional Ethics Review Boards of Nanjing University of Chinese Medicine, in accordance with the Guidelines (Ethics Registration no. 202205A007). Informed consent was obtained from all participants in the study.

## Supporting information




**Figure S1**: c‐Maf is associated with poor outcomes in MM patients and promotes MM cell proliferation. **(A)** The Human Protein Atlas database was used to analyze the abnormal upregulation of c‐Maf in multiple disease types, including MM. **(B & C)** Increased c‐Maf mRNA expression was positively associated with poor overall survival in TT2 **(B)** and TT3 **(C)** patient cohorts. **(D)** According to The Human Protein Atlas database, the expression of c‐Maf was higher in the t (14; 16) translocation type compared to the non‐translocation type in MM cell lines. **(E)** WB assay analysis showed a significant increase in c‐Maf expression in cell lines with chromosomal translocation compared to control cell lines. **(F)** The correlation between c‐Maf and Ki67 staining intensity in normal, non‐serious diagnosis, and serious diagnosis groups (reffered to Figure 1B). **(G)** WB method was used to check c‐Maf expression in WT and c‐Maf‐OE ARP1 and KMS28PE cells. **(H)** CCK‐8 assay demonstrated that c‐Maf‐OE MM cells had a stronger proliferation capacity compared to WT MM cells. **(I)** Cell apoptosis analysis showed that overexpression of c‐Maf weakened MM cell apoptosis induced by BTZ. **(J)** NovoPro database predicted the LLPS ability of c‐Maf. **(K)** Iupred2a database predicted the LLPS ability of c‐Maf. **(L)** The amino acid sequence of c‐Maf protein and the amino acids of IDRs are marked in yellow. Quantification of WB was obtained from three independent experiments. The data are expressed as the mean ± SD; *P* < 0.05 (*), *P* < 0.01 (* *) and *P* < 0.001 (* * *). WT, wild‐type; BTZ, bortezomib; WB, western blotting; MM, multiple myeloma.
**Figure S2**: The interaction between c‐Maf and the Mtbp/c‐Myc axis. DDX6 and ELP4 mRNA expression levels were detected in EV and c‐Maf‐EGFP transfected MM cell lines. **(B)** Agarose gel electrophoresis results of ChIP‐qPCR assay on the interaction between c‐Maf and the promoter region of Mtbp (reffered to Figure 3G). **(C)** Quantitative results of WB examination on Mtbp expression upon overexpression of c‐Maf and treatment with si‐Mtbp (reffered to Figure 3J). **(D)** Quantitative analysis of the effect of si‐Mtbp treatment on the stability of c‐Myc protein (reffered to Figure 5D). **(E)** Quantitative results of WB examination on c‐Maf, Mtbp, and c‐Myc expression upon overexpression of c‐Maf (reffered to Figure 5E). **(F)** IHC analysis of CDX model showed elevated expression of c‐Maf, RNA Pol II, Mtbp, c‐Myc, and Ki67 in c‐Maf‐EGFP group compared to EV group, which was inhibited in c‐Maf‐MUT‐EGFP group. **(G)** IF assay of the CDX model exhibited that induction of c‐Maf promoted co‐localizaion of c‐Maf and RNA Pol II, while c‐Maf‐MUT impaired this co‐localizaion. Scale bar: 36.8 µm. Quantification of WB was obtained from three independent experiments. The data are expressed as the mean ± SD; *P* < 0.05 (*), *P* < 0.01 (* *) and *P* < 0.001 (* * *). CDX, cell line‐derived xenograft; WB, western blotting; IHC, immunohistochemistry.
**Figure S3**: BBA fails to inhibit c‐Maf expression in MM cells. Predicted structure of c‐Maf by Alphafold (AlphaFold Protein Structure Database (ebi.ac.uk). **(B)** WB analysis showed that BBA exhibited no effect on the protein level of c‐Maf. **(C)** Quantitative results from the WB assay showed changes in c‐Maf expression after treatment with BBA. Quantification of WB was obtained from three independent experiments. WB, western blotting.

## Data Availability

All data supporting this study's findings are available from the corresponding authors upon reasonable request. Chromatin immunoprecipitation and next‐generation sequencing are available at GEO under accession number GSE264392. Mass spectrometry (MS) data can be found here: the Proteome Xchange Consortium: PXD051828.
