# Supplementary material for: Targeting the Liquid–Liquid Separation Region of c‐Maf for Treating Chromosomal Translocations in Multiple Myeloma
Source: MedComm (2020). 2025 Nov 11;6(11):e70464. doi: 10.1002/mco2.70464 (PMC12606047; doi:10.1002/mco2.70464)
Supplement: Supplementary file 1 — Figure S1: c‐Maf is associated with poor outcomes in MM patients and promotes MM cell proliferation. (A) The Human Protein Atlas database was used to analyze the abnormal upregulation of c‐Maf in multiple disease types, including MM. (B & C) Increased c‐Maf mRNA expression was positively associated with poor overall survival in TT2 (B) and TT3 (C) patient cohorts. (D) According to The Human Protein Atlas database, the expression of c‐Maf was higher in the t (14; 16) translocation type compared to the non‐translocation type in MM cell lines. (E) WB assay analysis showed a significant increase in c‐Maf expression in cell lines with chromosomal translocation compared to control cell lines. (F) The correlation between c‐Maf and Ki67 staining intensity in normal, non‐serious diagnosis, and serious diagnosis groups (reffered to Figure 1B). (G) WB method was used to check c‐Maf expression in WT and c‐Maf‐OE ARP1 and KMS28PE cells. (H) CCK‐8 assay demonstrated that c‐Maf‐OE MM cells had a stronger proliferation capacity compared to WT MM cells. (I) Cell apoptosis analysis showed that overexpression of c‐Maf weakened MM cell apoptosis induced by BTZ. (J) NovoPro database predicted the LLPS ability of c‐Maf. (K) Iupred2a database predicted the LLPS ability of c‐Maf. (L) The amino acid sequence of c‐Maf protein and the amino acids of IDRs are marked in yellow. Quantification of WB was obtained from three independent experiments. The data are expressed as the mean ± SD; P < 0.05 (*), P < 0.01 (* *) and P < 0.001 (* * *). WT, wild‐type; BTZ, bortezomib; WB, western blotting; MM, multiple myeloma. Figure S2: The interaction between c‐Maf and the Mtbp/c‐Myc axis. DDX6 and ELP4 mRNA expression levels were detected in EV and c‐Maf‐EGFP transfected MM cell lines. (B) Agarose gel electrophoresis results of ChIP‐qPCR assay on the interaction between c‐Maf and the promoter region of Mtbp (reffered to Figure 3G). (C) Quantitative results of WB examination on Mtbp expression upon overexpr [file MCO2-6-e70464-s001.docx]

**Targeting the Liquid-Liquid Separation Region of c-Maf for Treating Chromosomal Translocations in Multiple Myeloma**

**Running title:** Targeting c-Maf LLPS in multiple myeloma

Ze Wang^1,2#^, Mengjie Guo^2#^, Xichao Yu^2#^, Yi Sun^2^, Haowen Bai^2^, Lianxin Zhou^2^, Zihao Liu^2^, Hongming Huang^3^, Chong Wang^4^, Hong Liu^3^, Chunyan Gu^1,2*^, Ye Yang^2*^

**Affinitations:**

^1^ Nanjing Hospital of Chinese Medicine affiliated with Nanjing University of Chinese Medicine, Nanjing, China;

^2^School of Medicine, Nanjing University of Chinese Medicine, Nanjing, 210023, China;

^3^ Department of Hematology, The Affiliated Hospital of Nantong University, 20 Xisi Road, Nantong 226001, China;

^4^ Department of Hematology, the First Affiliated Hospital of Zhengzhou University, Zhengzhou 450052, China.

**#** These authors contributed equally.

***Corresponding authors**

Chunyan Gu, E-mail: guchunyan@njucm.edu.cn

Ye Yang, E-mail: yangye876@sina.com

Tel: +86 02585811597

Address: Nanjing University of Chinese Medicine, 138 Xianlin Road, Nanjing 210023, China.

**4532 words, 7 figures, 3 supplement figures, 2 tables, 48 references.**

**Supplementary Methods and Materials**

**Antibodies and reagents**

The primary antibodies used in this study were as follows: c-Maf (55013-1-AP, ProteinTech Group, China), GAPDH (60004-1-Ig, ProteinTech Group, China), GFP (50430-2-AP, ProteinTech Group, China), CD138 (10593-1-AP, ProteinTech Group, China), Ki67 (AF0198, Affinity, USA), DYKDDDDK (66008-4-Ig, ProteinTech Group, China), and Mtbp (28356-1-AP, ProteinTech Group, China). The secondary antibodies used were Goat anti-Rabbit IgG(H+L) HRP (sc-2005, Santa cruz, USA) and Goat anti-Mouse IgG(H+L) HRP (sc-2004, Santa cruz, USA). BTZ was purchased from Selleck Chemicals (Houston, TX), while Puromycin (60210ES25), Polybrene (40804ES75), and Lipofectamine Reagent (40802ES03) were purchased from Yeasen Biotechnology (Shanghai) Co., Ltd. Dexamethasone was purchased from Beijing Inokey Technology Co., Ltd. The Pierce™Direct Magnetic IP/Co-IP Kit was purchased from Thermo Fisher Scientific (Massachusetts). BBA was kindly gifted from Professor Li Nianguang's laboratory.

**Cell lines and cell culture**

The human MM cell lines, ARP1, MM1.S, H929, and KMS28PE, were cultured in RPMI 1640 (05-065-1A, Bioind, Israel). HEK293 was cultured in DMEM (01-052-1ACS, Bioind, Israel). Both RPMI 1640 and DMEM were supplemented with 10% fetal bovine serum (FBS, A6901, Introvigen, USA) and 1% penicillin/streptomycin (03-031-1B, Biological Industries). The cells were cultured at 37℃ in a humidified incubator supplied with 5% CO_2_.

**Plasmids and transfection**

The plasmid containing human c-Maf cDNA was purchased from Shanghai Jikai Biotechnology Co., Ltd. The plasmid containing human RNA Pol II cDNA was purchased from Wuhan Miaoling Biotechnology Co., Ltd. The cDNA sequence of c-Maf was cloned into the lentiviral vector GV358 and prokaryotic vector pET-28a, while the cDNA sequence of RNA Pol II was cloned into the eukaryotic vector pCMV and prokaryotic vector pET-28a. The expression vector (c-Maf cDNA /RNA Pol II cDNA) and packaging vector (PSPA, PMD2G) were co-transferred into HEK293 cells by Lipofectamine transfection reagent (YEASEN, Shanghai). After 48 h of cultivation, the virus supernatant was collected and transfected into MM cells. Then, transfected MM cells were selected by puromycin resistance. Transduction efficiency was validated by western blotting (WB).

**Cell proliferation, cell cycle and apoptosis assays**

We adjusted the cell density to 4,000 cells/well (ARP1 EV/c-Maf-OE) and 6,000 cells/well (KMS28PE EV/c-Maf-OE) on a 96-well plate. The purpose of this experiment was to measure cell proliferation and viability using the Cell Counting Kit-8 (CCK-8) assay at 24, 48, and 72 h. The absorbance was detected at 450 nm using a microplate reader (Varioskan LUX, Thermo). For colony formation, 1×10^4^ MM cells were seeded in a 24-well plate with 0.5 mL of a mixture of 0.33% agar and RPMI 1640 plus 10% FBS. We replaced the medium twice per week for 14 days. The colonies were imaged and counted using Image J until the number of cells was >40, which were considered to be clone-forming colonies. For the cell cycle assay, cells were resuspended in 70% ethanol at -20℃ overnight. Ribonuclease A (10405ES03, YEASEN) was added 1 h before resuspending the cells. The cells were then stained with propidium iodide (PI) (c0080, Solarbio LIFE SCIENCES) for 15 min at room temperature. Flow cytometry was performed using the Guava easyCyteTM system from Merck Millipore in Germany. For cell apoptosis assay, cells were treated with BTZ. We measured apoptosis by labeling cells with APC conjugated AnnexinV (B317159; Biolegend) in combination with PI. The resulting cells were analyzed using GuavaSoft, and the data were processed through FlowJo.

**Co-immunoprecipitation (Co-IP)**

Co-IP was performed using a Pierce Direct Magnetic IP/Co-IP kit (Thermo Scientific), following the instructions provided by Thermo Scientific.

**Microscale thermophoresis (MST)**

To determine if the screened compound interacts with the c-Maf protein, we used a MonolithTM RED-NHS second generation protein labelling kit (NanoTemper Germany) to fluorescently label 10 µM of purified c-Maf protein. We then mixed various concentrations of the drug with an equal concentration of c-Maf protein and incubated the mixture for 5 min at room temperature. The mixture was then aspirated with a capillary tube and the binding capacity was measured using a new generation biomolecular interaction assay (NanoTemper Germany). The Kd values were calculated using analytical software.

**MM cell line-derived xenograft (CDX) model**

We subcutaneously injected 1×10^6^ pCDH-EV (EV), c-Maf-EGFP and c-Maf-IDR-mutation-EGFP (c-Maf-MUT-EGFP) cells into left and right abdominal flank of 6-8 weeks old SCID/NOD mice (n = 18), respectively. Calipers were used to measure tumor diameter three times weekly. When the tumor size reached 15 mm, we sacrificed the mice with intraperitoneal injection of pentobarbital sodium anesthesia. Afterwards, the tumors were collected, weighed, and photographed.

**MM Patient-Derived Tumor Xenograft (PDX) model**

The biopsy sample was taken from tumor tissue excised from MM patient at Nanjing Hospital of Chinese Medicine affiliated with Nanjing University of Chinese Medicine. The tissue was then cut into 2.5×2.5×2.5 mm^3^ pieces and transplanted subcutaneously into 6-week-old NOD/SCID male mice (n = 6 mice in each group) under anesthesia with 1% sodium pentobarbital. Once the tumors reached a size of 500 mm3, they were divided into 2.5×2.5×2.5 mm^3^ pieces and implanted into the NOD/SCID mice again. This process was repeated three times to ensure stable tumor growth. When the tumors reached a size of 100-150 mm^3^, the mice were randomly divided into four groups: control (Ctrl), benzoyl benzoic acid treatment (BBA, 5 mg/kg, i.g.), BTZ treatment (BTZ, 1 mg/kg, i.p.), and BBA+BTZ combination groups. Single treatment was administered every three days, while combination treatment was administered every other day.

**5TMM3VT mouse model**

5TMM3VT mouse myeloma cells (1 × 10^6^) were injected through the tail vein into C57BL/KaLwRij mice from Harlan Laboratories (n = 9 per group). The mice were randomly divided into four groups (Ctrl, BBA, BTZ and BBA+BTZ combination groups). On the third day after modeling, oral administration of PBS, BBA (5 mg/kg) and BTZ (1 mg/kg) was performed twice a week, while combination therapy was administered every other day.

**Fluorescence recovery after photobleaching (FRAP)**

FRAP experiments were conducted on ARP1 and KMS28PE cell lines transfected with c-Maf EGFP fusion plasmid by using a confocal microscope (TCS SP8, Leica, Germany). Tornado mode with 488 nm laser at 80% laser power was used to perform photobleaching for 5 s. Free running mode with 488 nm laser was applied to monitor EGFP fluorescence recovery at approximately 5-second intervals. Also, the fluorescence of unbleached sites in the same view was measured as a control. The signal was represented as the ratio of the post-bleaching fluorescence signal to the pre-bleaching fluorescence signal.

***In vitro* droplet assay**

To enrich and purify the protein, we transformed plasmids containing the sequence for the EGFP fusion protein into *E. coli* BL21 cells. Then, a fresh colony of BL21 cells with an OD600 of 0.6 was induced with 0.5 mM IPTG for 18 h. The precipitate was collected by centrifugation at 6,000 rpm for 15 min and then diluted with Buffer A solution (pH = 7.5, 500 mM NaCl, 500 mM HEPES, 5 mM imidazole, 5% glycerol), protease inhibitor added, followed by homogenized at 800-900 bar for 15 min. After centrifugation and collecting the supernatant, the protein solution was filtered through a 0.45 μm aqueous membrane. Afterwards, an AKTA protein separation and purification platform (AKTA pure L, Cytiva, USA) was employed to elute and obtain the target protein by Buffer B (pH = 8, 50 mM NaCl, 20 mM Tris). Then, the purity and concentration of proteins were determined by coomassie-stained gels and GF Buffer (pH = 8, 50 mM NaCl, 20 mM Tris). The purity was concentrated, quantified and stored. For the droplet assay, proteins were diluted with Buffer A to 5, 10, and 20 μM. A Leica TCS SP8 confocal microscope (TCS SP8, Leica, Germany) was used to observe the number and size of droplets formed by LLPS of c-Maf. Subsequently, PEG-8000 with final concentrations of 2.5%, 5% and 10% was used to induce *in vitro* phase separation of c-Maf protein. Meanwhile, NaCl with final concentrations of 25, 50, 100, and 200 mM, as well as 1,6-hexanediol with final concentrations of 5%, 10% and 20% were used to induce depolymerization of c-Maf protein. Then, the mixture was gently mixed and let stand for 2 min. A uniform drop was added to a confocal dish and imaging was captured through a Leica TCS SP8 confocal microscope.

**Screening of small molecule compounds targeting c-Maf**

Commercial databases containing over 3 million small molecules (Specifications, Chembridge, Enamine, Hybrid) were utilized to screen for compounds targeting c-Maf by employing the Glide module of Schr ö dinger software. Each molecule was evaluated in 30 conformations. We selected compounds with a Glide Gscore below -5 and critical hydrogen bonding with key amino acids H184 and H186. Ultimately, 6 compounds, including BBA, were chosen.

**Supplementary Figures**

**
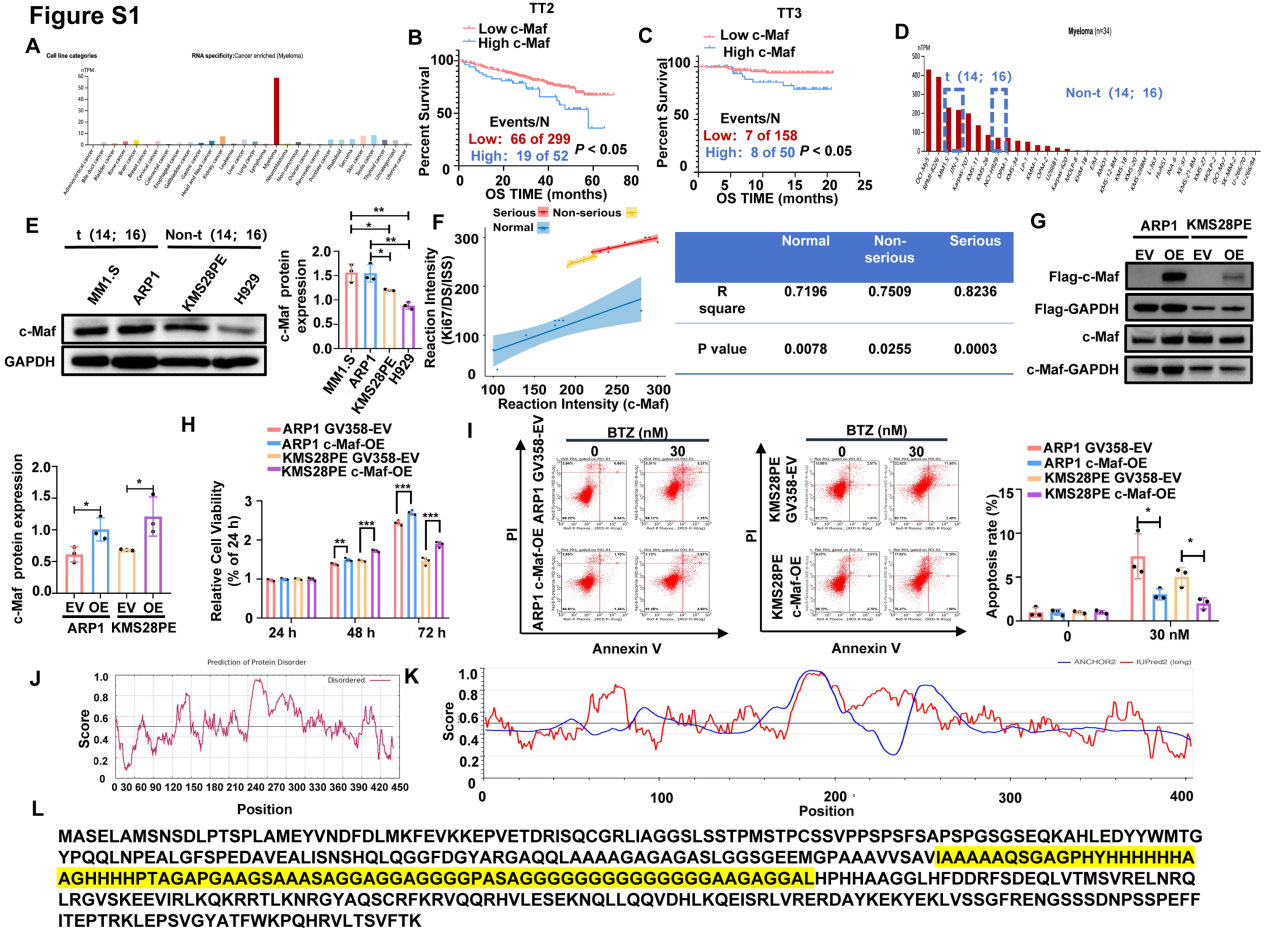
**

**Figure S1 c-Maf is associated with poor outcomes in MM patients and promotes MM cell proliferation.**

**(A)** The Human Protein Atlas database was used to analyze the abnormal upregulation of c-Maf in multiple disease types, including MM. **(B & C)** Increased c-Maf mRNA expression was positively associated with poor overall survival in TT2 **(B)** and TT3 **(C)** patient cohorts. **(D)** According to The Human Protein Atlas database, the expression of c-Maf was higher in the t (14; 16) translocation type compared to the non-translocation type in MM cell lines. **(E)** WB assay analysis showed a significant increase in c-Maf expression in cell lines with chromosomal translocation compared to control cell lines. **(F)** The correlation between c-Maf and Ki67 staining intensity in normal, non-serious diagnosis, and serious diagnosis groups (reffered to Figure 1B). **(G)** WB method was used to check c-Maf expression in WT and c-Maf-OE ARP1 and KMS28PE cells. **(H)** CCK-8 assay demonstrated that c-Maf-OE MM cells had a stronger proliferation capacity compared to WT MM cells. **(I)** Cell apoptosis analysis showed that overexpression of c-Maf weakened MM cell apoptosis induced by BTZ. **(J)** NovoPro database predicted the LLPS ability of c-Maf. **(K)** Iupred2a database predicted the LLPS ability of c-Maf. **(L)** The amino acid sequence of c-Maf protein and the amino acids of IDRs are marked in yellow. Quantification of WB was obtained from three independent experiments. The data are expressed as the mean ± SD; *P* < 0.05 (*), *P* < 0.01 (* *) and *P* < 0.001 (* * *). WT, wild-type; BTZ, bortezomib; WB, western blotting; MM, multiple myeloma.

**
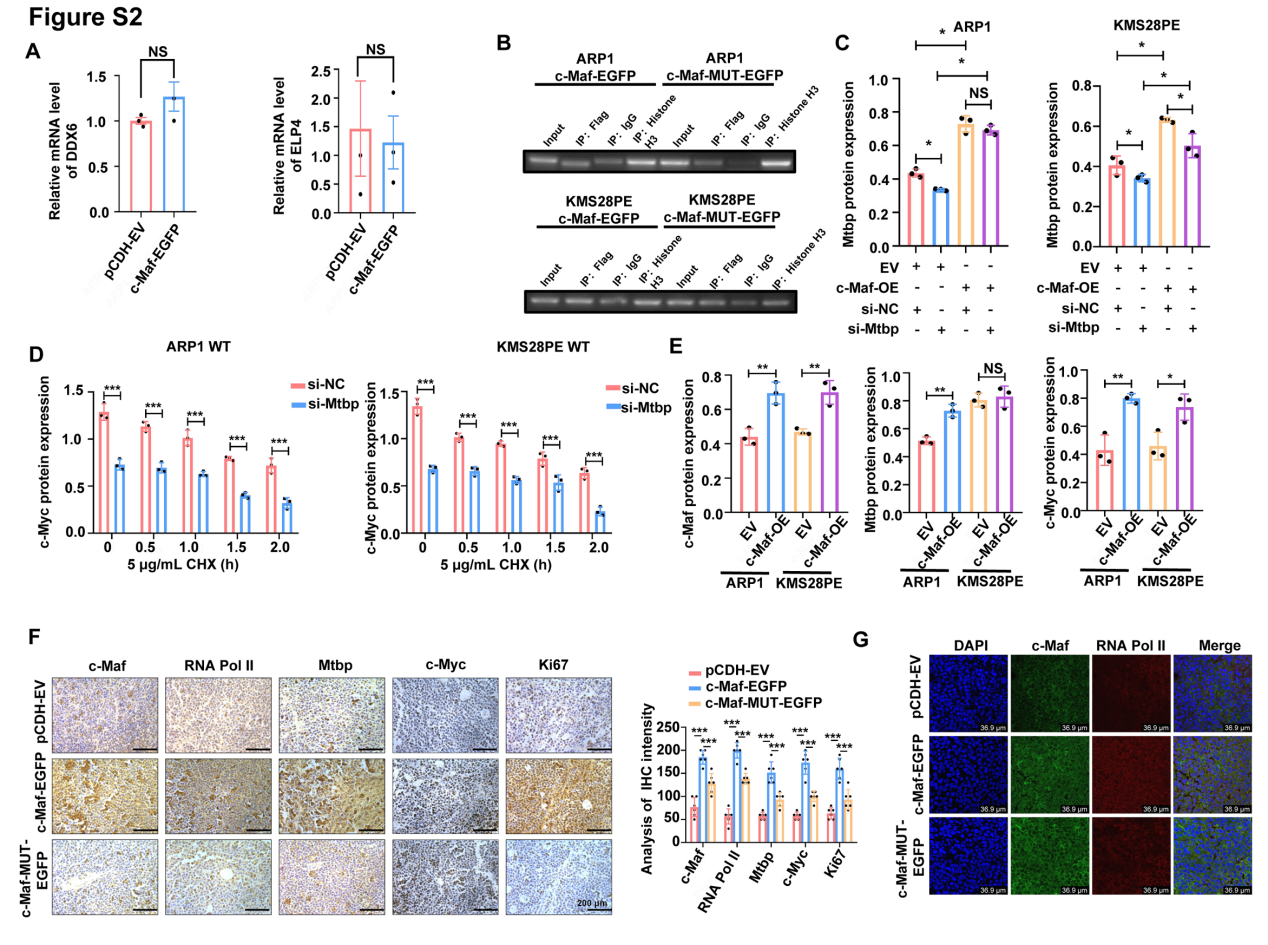
**

**Figure S2 The interaction between c-Maf and the Mtbp/c-Myc axis.**

1. DDX6 and ELP4 mRNA expression levels were detected in EV and c-Maf-EGFP transfected MM cell lines. **(B)** Agarose gel electrophoresis results of ChIP-qPCR assay on the interaction between c-Maf and the promoter region of Mtbp (reffered to Figure 3G). **(C)** Quantitative results of WB examination on Mtbp expression upon overexpression of c-Maf and treatment with si-Mtbp (reffered to Figure 3J). **(D)** Quantitative analysis of the effect of si-Mtbp treatment on the stability of c-Myc protein (reffered to Figure 5D). **(E)** Quantitative results of WB examination on c-Maf, Mtbp, and c-Myc expression upon overexpression of c-Maf (reffered to Figure 5E). **(F)** IHC analysis of CDX model showed elevated expression of c-Maf, RNA Pol II, Mtbp, c-Myc, and Ki67 in c-Maf-EGFP group compared to EV group, which was inhibited in c-Maf-MUT-EGFP group. **(G)** IF assay of the CDX model exhibited that induction of c-Maf promoted co-localizaion of c-Maf and RNA Pol II, while c-Maf-MUT impaired this co-localizaion. Scale bar: 36.8 μm. Quantification of WB was obtained from three independent experiments. The data are expressed as the mean ± SD; *P* < 0.05 (*), *P* < 0.01 (* *) and *P* < 0.001 (* * *). CDX, cell line-derived xenograft; WB, western blotting; IHC, immunohistochemistry.

**
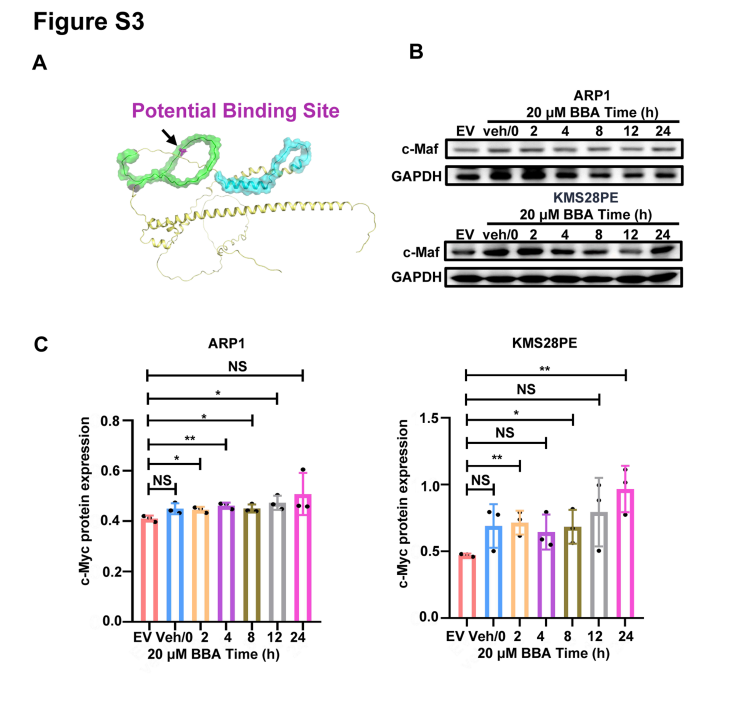
**

**Figure S3 BBA fails to inhibit c-Maf expression in MM cells.**

1. Predicted structure of c-Maf by Alphafold (AlphaFold Protein Structure Database (ebi.ac.uk). **(B)** WB analysis showed that BBA exhibited no effect on the protein level of c-Maf. **(C)** Quantitative results from the WB assay showed changes in c-Maf expression after treatment with BBA. Quantification of WB was obtained from three independent experiments. WB, western blotting.
